# The Role of Brief Global Cognitive Tests and Neuropsychological Expertise in the Detection and Differential Diagnosis of Dementia

**DOI:** 10.3389/fnagi.2021.648310

**Published:** 2021-06-10

**Authors:** Marianna Riello, Elena Rusconi, Barbara Treccani

**Affiliations:** Department of Psychology and Cognitive Science, University of Trento, Trento, Italy

**Keywords:** aging, dementia, cognitive assessment, screening tools, psychometric testing, cognitive impairment, cognitive decline, neuropsychology

## Abstract

Dementia is a global public health problem and its impact is bound to increase in the next decades, with a rapidly aging world population. Dementia is by no means an obligatory outcome of aging, although its incidence increases exponentially in old age, and its onset may be insidious. In the absence of unequivocal biomarkers, the accuracy of cognitive profiling plays a fundamental role in the diagnosis of this condition. In this Perspective article, we highlight the utility of brief global cognitive tests in the diagnostic process, from the initial detection stage for which they are designed, through the differential diagnosis of dementia. We also argue that neuropsychological training and expertise are critical in order for the information gathered from these omnibus cognitive tests to be used in an efficient and effective way, and thus, ultimately, for them to fulfill their potential.

## Introduction

With age comes wisdom, sometimes, but psychophysical decline always does. A decrease in the efficiency of cognitive functioning is among the typical age-related changes (Singh-Manoux et al., 2012). When such decline is so significant as to markedly interfere with social, occupational or domestic functioning, it is considered pathological and is referred to as *dementia*.

In common parlance, dementia has somehow become synonymous with Alzheimer Disease (AD) and with its most notorious symptom, that is, memory loss. However, according to the most recent version of the Diagnostic and Statistical Manual of Mental Disorders (DSM-5; American Psychiatric Association, 2013), a memory impairment is no longer necessary to diagnose dementia. A diagnosis of Major Neurocognitive Disorder (i.e., the term that replaced the term dementia in the DSM-5) requires evidence of significant cognitive decline from a previous level of performance in one or more cognitive domains, which may or may not include memory, as well as interference of the cognitive deficit(s) with independence in daily activities, lack of exclusive occurrence in the context of a delirium, and lack of a better explanation based on another mental disorder. Similarly, the less severe form of cognitive impairment with no major repercussions on daily life, known as mild cognitive impairment (MCI, or Mild Neurocognitive Disorder in the DSM-5), can present itself with deficits that involve only (or predominantly) memory (i.e., the amnestic MCI), but can also involve another cognitive domain, or more than one cognitive domain, either with or without memory impairment. Indeed, besides memory loss and memory-related deficits (e.g., misplacing things and difficulties in keeping track of things, being confused about time and place), among the common signs and symptoms of dementia there can also be found: receptive and productive language problems, difficulties in solving problems and carrying out familiar daily tasks, difficulties in planning and organizing, changes in judgment or decision-making functions, difficulties in understanding visual images and spatial relationships, difficulties in perceptual-motor coordination, inappropriate behavior and changes in mood or personality, withdrawal from work or social activities (e.g., Alzheimer’s Disease International, n.d.).

In some cases, the aging population is screened for detection of these signs of dementia (cf., Foster et al., 2019). However, in most cases, it is the aging individual’s own awareness of a subjective decrease in cognitive functioning (leading to increased difficulty or inability to cope with daily activities) or the detection of impoverished performance by a close observer that triggers an initial examination by a general practitioner (Moore et al., 2018). The general practitioner will then decide whether to proceed with further examinations and/or refer the patient for a more in-depth examination to a specialist practitioner, who will reach a definite diagnosis based on history, examination and objective assessments (Hugo and Ganguli, 2014; Falk et al., 2018).

### Brief Global Cognitive Tests

Tools used in the diagnostic process of dementia include informant questionnaires on both cognitive decline and autonomy in activities of daily living, along with tests of cognitive function providing an objective assessment of cognitive impairment. Typically, the latter are brief global cognitive function tests, that is, paper-and-pencil neuropsychological tests with short administration times aimed at assessing an individual’s general mental status (Arevalo-Rodriguez et al., 2015). According to recent reviews of the literature (Hwang et al., 2019; Razak et al., 2019), the most popular global cognitive function tests used to this end are: the Mini Mental State Examination (MMSE), the Mini Cog Test, the Montreal Cognitive Assessment (MoCA), the General Practitioner Assessment of Cognition (GPCOG), and the Clock Drawing Test (CDT; see Table 1).

**TABLE 1:** Global cognitive tests used in the diagnosis of dementia: sub-scores, items and specific skills or functions required for their correct execution (General skill: Verbal auditory comprehension and working memory).

Test	Items		Specific skills or functions
**Clock Drawing Test** (CDT; Hubbard et al., 2008)	Drawing of a clock from memory with hands indicating a specific time (e.g., 10 past 11; Watson et al., 1993)		Visuo-constructive and visuo-spatial skills, semantic memory, executive functions, and abstract thinking
**Mini Cog** (Borson et al., 2000)	Clock drawing		See CDT

	Immediate recall of three unrelated words		Speech perception and production, and working memory
	Delayed recall of the three words		Episodic verbal long-term memory

**General Practitioner Assessment of Cognition** (GPCOG, Brodaty et al., 2006)	Cognitive section	Clock drawing	See CDT
		Time orientation	Temporal orientation
		Delayed recall of a name and address	Episodic verbal long-term memory
		Report of a recent piece of news	Temporal and spatial orientation, and episodic and semantic memory
	
	Questionnaire for the caregiver requiring a comparison of the testee’s current cognitive and behavioral profile to that of 5–10 years ago (only in case of a score between 5 and 8 in the cognitive section)		All functions and skills contributing to global cognitive and behavioral profile

**Mini-Mental State Examination** (MMSE; Folstein et al., 1975)	Orientation (time and space)		Temporal and spatial orientation
	Repetition of three unrelated words		Speech perception and production, and working memory
	Delayed recall of the three words		Episodic verbal long-term memory
	Calculation (serial subtraction of 7 from 100 (five times) or spelling of “WORLD” backward		Calculation, working memory, selective attention, and sustained attention
	Naming of a wristwatch and a pencil		Semantic memory, (phonological) lexical retrieval
	Repetition of a sentence		Speech perception and production, and working memory
	Execution of three simple commands		Planning, prospective memory (ability to remember to perform a planned action), and ideomotor praxis
	Read and execute commands		Reading, prospective memory, and ideomotor praxis
	Write a sentence with a noun and a verb		Writing and syntactic skills, and executive functions (verbal fluency)
	Constructional praxis: Copy of a picture		Visuo-constructive and visuo-spatial skills

**Montreal Cognitive Assessment** (MoCA; Nasreddine et al., 2005)	Visuospatial functions	Copy of a picture (a cube)	Visuo-constructive and visuo-spatial skills
		Clock drawing	See CDT
	
	Executive functions	Alternating trial making test	Visual search and scanning, psychomotor speed, executive attention (selective attention, divided attention, response inhibition, and attention shifting), and planning
		Abstraction: Recognition of what pairs of objects have in common	Semantic memory, abstract reasoning, and problem solving
		Phonemic fluency task*	Verbal fluency
	
	Language	Naming: denomination of three unfamiliar animals*	Semantic memory, and phonological lexical retrieval
		Repetition of two sentences*	Speech perception and production, and working memory
	
items marked with an asterisk (*) are those used to calculate the Moca VLOM subscore	Attention and working memory	Target detection (letter A in a list of letters) using tapping	Sustained attention, inhibitory control (response inhibition), and ideomotor praxis
		Calculation: Serial subtraction of 7 from 100 (five times)	Calculation, working memory, selective attention, and sustained attention
		Forward and backward digit span	Working memory
			
	
	Memory	Repetition of five unrelated words	Speech perception and production, and working memory
		Delayed recall of the three words*	Episodic verbal long-term memory
	
	Orientation	Orientation (time and space)*	Temporal and spatial orientation

*CDT: *Duration*, 2 min; *Maximum score*, 7 (Watson et al., 1993); *Aim*, detection of early signs of dementia. Mini Cog: *Duration*, 7–8 min; *Maximum score*, 30; *Aim*, detection of dementia. GPCOG: *Duration*, 4 min (two additional minutes for the questionnaire). *Maximum score*: Cognitive section, 9; questionnaire, 6; *Aim*, detection of dementia (family physicians, general practitioners). MMSE: *Duration*, 3–4 min; *Maximum score*, 5; *Aim*, mainly used to detect moderate-to-severe forms of dementia. MoCA: *Duration*, 10 min; *Maximum score*, 30; *Aim*, mainly used to detect MCI (especially suitable for people scoring above 24 on the MMSE). The list of cognitive functions/skills required by test items are based on definitions and cognitive analyses included in the test original descriptions but also on those provided by pivotal references (cognitive psychology and neuropsychology books and papers) on the cognitive constructs under examination (e.g., Denes and Pizzamiglio, 1999; Lezak et al., 2012; Baddeley et al., 2020). For example, we use the term “working memory” in reference to the cognitive processes that allow people to temporally store small amounts of information (e.g., the test instructions, a short list of words or numbers) over brief delays but also to manipulate such information (Baddeley et al., 2020). Accordingly, working memory tasks include typical short-term memory tasks (such as verbal serial recall), but also tasks requiring both the temporary storage of small amounts of (either verbal or visuospatial) material and additional processes that allow the manipulation of this material, such as those usually referred to as attentional shifting, updating, and selective attention (e.g., changing the order of the to-be-recalled items, updating the contents of working memory, inhibiting irrelevant information; Gathercole et al., 2019).*

These brief, global cognitive tests are often defined as *cognitive screening tools* as they are typically used at a population screening stage to detect potential cognitive impairment that may raise the suspicion of dementia. In many handbooks for mental health practitioners, they are indeed presented as satisfying the requirements for optimal screening instruments, which include brief duration, good test-retest and inter-rater reliability, sampling of all major cognitive domains, and, last but not least, easiness of both administration and interpretation. It is generally claimed that any clinician, or health care professional, with *any level of training*, should be able to administer these tests and interpret their results, thanks to their clear cutoffs (Malloy et al., 1997; Larner, 2017), and they are indeed frequently administered and interpreted by non-specialist professionals (see, e.g., Arevalo-Rodriguez et al., 2015; Palm et al., 2016).

Even if designed as screening tools, these global cognitive tests are often also used at later diagnostic stages—in practice, most evidence about a person’s cognitive status is often gathered through these tests in all phases of the diagnostic process. However, clinical practice guidelines for dementia assessment warn examiners about such use of these tests and recommend that a comprehensive cognitive evaluation of the testee, including specific tests assessing different cognitive functions, be performed in order to reach a diagnosis (cf., Di Pucchio et al., 2018).

Here we present our perspective on the topic and argue that, even though a detailed neuropsychological assessment, coupled with a thorough clinical evaluation, does remain the gold standard for an accurate diagnosis, global cognitive tests can provide *specific* information and be actually very useful not only at a first, screening, stage of the diagnostic process, but also at the following stages, in which more refined information about a testee’s cognitive status is required. In our view, the training and neuropsychological expertise^1^ of the health care professional who uses these tests is what makes the difference. Results obtained through these tests can be extremely informative when read by professionals with an adequate knowledge of the cognitive functions that they aim to test. In contrast with what is usually held (i.e., that very little training is required for the use of these tests in the first, preliminary stages of the diagnostic process), we maintain that, in all the diagnostic phases in which they are used, they should be used by professionals with in-depth neuropsychological expertise. Such an expertise is critical for all the aspects connected with the use of these tests: the *selection* of the most appropriate test according to the purpose of the assessment (e.g., screening, diagnostic confirmation, differential diagnosis, and monitoring of symptom evolution), the setting (e.g., primary or secondary care) in which testing is undertaken and the population from which the testee is drawn, as well as aspects related with test *administration*, *scoring*, and both the *interpretation* and *communication* of results.

## The Role of Global Cognitive Tests and Neuropsychological Expertise in the Diagnostic Process

As any diagnostic process, the diagnosis of dementia can be divided into two main stages: detection of dementia (which ends with the confirmation of the initial diagnostic suspicion) and etiological/differential diagnosis. On the whole, this process is characterized by the use of different methods of investigation (e.g., colloquia, cognitive tests, and laboratory exams) and may last for an extended time window, spanning from the early detection of potential symptoms to the monitoring of symptom evolution and identification of etiological components.

### Detection of Dementia

Dementia may be inadequately recognized in primary care settings and it often goes undetected, at least when the symptoms are mild (Lang et al., 2017; Hwang et al., 2019). There are many reasons why elderly people and people who are close to them, or even the family physician, may not notice initial symptoms of dementia or may not judge such symptoms as needing assessment – for example, poor understanding of the difference between the memory decline due to normal aging and that observed in dementia (Ashford et al., 2007).

In light of this, several national and international consensus groups have promoted the routine screening of at-risk populations (e.g., people older than 65; Hwang et al., 2019). However, the need for an early diagnosis and early screening at the population level is not universally acknowledged (e.g., Chambers et al., 2017) and some panels of experts actually recommend not to screen individuals when the individuals themselves, or people close to them, do not express concerns about the presence of cognitive impairment (e.g., Lin et al., 2013).

Advocates of routine screening maintain that most of the criteria endorsed by the World Health Organization to trigger disease screening (Wilson and Jungner, 1968) are fulfilled by dementia and that missed or delayed diagnoses may lead to lost treatment opportunities and increase both patient and caregiver burden (e.g., Bradford et al., 2009). In contrast, opponents argue that it has yet to be demonstrated that any of the available treatment for the most common subtypes of dementia (AD, in the first place) are more beneficial when applied in a pre-symptomatic phase than at later (symptomatic) stages (e.g., Larner, 2017). Moreover, routine screening comes with potential harms, which do not only include economic issues (i.e., financial costs associated with screening), but also a higher probability of misdiagnosis. Highly sensitive tests, such as those recommended for screening purposes, carry a high risk of false positive diagnoses. According to the Canadian Task Force on Preventive Health Care (Pottie et al., 2016), for example, one in eight to ten individuals without cognitive impairment screened for dementia and MCI are incorrectly classified using the MMSE. In turn, these misdiagnoses entail psychological costs: a positive result at a screening test can be mistaken for a diagnosis of dementia which would, at best, generate anxiety in the positive screened individuals and their relatives, but could also trigger depression, loss of status, loss of employment, stigmatization, institutionalization, and loss of independence (e.g., the individual may stop driving or making independent financial and healthcare decisions; Chambers et al., 2017).

These potential harms, however, could be avoided if screening occurs under the supervision of an expert acknowledging the limitations of a positive outcome (i.e., that screening of dementia is not equivalent to a diagnosis), knowing both the actual probability of dementia/MCI in case of a positive screening outcome and how to appropriately ***communicate*** the implications of such an outcome to the screened individuals and their families. Adequate communication (i.e., communication made by an expert) of the outcomes of the evaluation is actually needed even when such outcomes are negative. Data from memory/cognitive clinics specialized in the diagnosis of dementia show that around 50% of older people who go to their physician or directly to these clinics with complaints about cognitive impairments turn out not to have either dementia or MCI (e.g., Iliffe and Wilcock, 2017). Individuals with only subjective cognitive impairment need to be reassured and informed about the possible changes in cognition that occur with normal aging. A degree of (neuro)psychological expertise of the professional who communicates the outcome is thus always highly desirable, whether it is positive or negative.

Cognitive impairment in individuals with subjective complaints should not obviously be ruled out solely because a person obtained a normal score on a screening cognitive test. Indeed, regardless of what raises the suspicion of cognitive impairment (i.e., a positive result in a routine population screening, self- or other-referral), it normally triggers a first general, comprehensive evaluation aimed to investigate this suspicion and rule out reversible forms of cognitive decline. Usually, at this (confirmatory) stage, the assessment of a testee’s general mental status is performed in non-specialist settings and with the same global cognitive tests as those used in population routine screenings. As previously mentioned, such tests are usually described as easy-to-administer tools and thus they are seen as suitable to be administered by general health professionals in typical primary, residential and acute care settings (i.e., to community-dwelling older adults or elders in nursing homes and hospitals; Hwang et al., 2019).

Very recently, however, attention has been drawn to the need of appropriate training for the ***administration*** of these apparently simple tests following the decision taken by the creator and copyright owner of the MoCA, Dr. Ziad Nasreddine, to make it only available to certified trainers (Nasreddine, n.d.). According to him, this move will reduce variability and ensure the highest accuracy: among the MoCA protocols he reviewed, he found many obvious testing inaccuracies (i.e., it was clear that the examiner had not followed the test instructions) or implausible test-retest variability (e.g., a change of as many as five points in the same individual over a few weeks; Aleccia, 2019).

High measurement errors, inter-rater, intra-rater, and test-retest variability are indeed the most important limitations of this kind of tests (Clark et al., 1999). Standardized administration protocols and related training may obviously reduce such errors and variability by increasing measurement accuracy (c.f., e.g., Kaplan and Saccuzzo, 2005). Neuropsychological expertise can further improve measurement accuracy. It has been shown that individual scores in scales aimed at measuring cognitive impairment may dramatically differ when they are administered by non-specialist health care professionals from when they are administered by specialists, even if non-specialists have undergone a specific training on the administration procedures (e.g., Fabrigoule et al., 2003). There might be several reasons for that. Professionals with neuropsychological expertise may know how to foster cooperation and maximal effort during testing, thanks to their experience in structured colloquia and neuropsychological test administration. Moreover, *knowledge of the psychological constructs* under investigation allows the examiner to make appropriate choices on how to manage the test administration in all its aspects, such as the choice of the most suitable place where to administer the test and of the allowable adjustments of the administration protocol that may be implemented to deal with unexpected or non-standard conditions.

For example, the CDT is considered to be very easy to administer. Nevertheless, its administration requires knowledge of all cognitive functions involved in the execution of the test. The examiner should know that it aims to evaluate constructional and visuo-spatial skills but also several other cognitive functions, such as semantic-memory processes (e.g., those involved in recalling both the visual structure of a clock and the number symbols of the hours on the clock), and should acknowledge that the evaluation of these aspects is of great importance. In light of this, the examiner should check, for example, that the testing room does not contain any real-world model of a clock (e.g., wall clocks, pictures of a clock).

Similarly, an examiner administering the calculation item of the MMSE (see Table 1) should be fully aware that such item does not aim to evaluate basic mathematical skills but mainly taps attentional and working memory process. Therefore, the examiner should avoid prompting testees when they hesitate during the sequence of calculations by reminding them the results of a previous subtraction, in order to urge them to continue with the next one. Likewise, the read-and-execute-commands item does not only evaluate basic written language comprehension skills, but also prospective memory and high-level verbal ability: the testee has to remember to perform the action contained in the written command that will be presented right after the instructions, which thus have to be understood without knowing the specific content of the command to be executed.

Finally, in-depth knowledge of the cognitive functions being evaluated by a given test or test item may allow the examiner to make the right choice when slight adjustments of the administration protocol are needed because, for example, of a testee’s physical disability (e.g., vision or hearing impairments, which are not unusual in elderly people; cf. Turner et al., 2001).

In the light of this, clinical guidelines recommend that whenever test administrators with high neuropsychological expertise are unavailable (e.g., tests have to be administrated by general health professionals), overall supervision by people with expertise be always granted (e.g., Ballard et al., 2015).

Such a supervision appears all the more critical when test ***scoring***, rather administration, is concerned. Even very brief tests demand *ad hoc* training for test scoring. For example, scoring the CDT is not intuitive. Correct performance on this test reflects the integrity of many interdependent cognitive functions and only raters with both training and experience are aware of the many possible errors that a testee can make. Common mistakes of naïve CDT scorers include not taking into account errors in the spacing of numbers on the clock face (Lorentz et al., 2002) or possible switches between different numerical codes (e.g., from Arabic to Roman). Such inconsistencies may appear trivial to the untrained scorer but they actually signal problems in visual-spatial and executive functions (just as errors in hand placement), which manifest themselves in poor monitoring of the spatial relations between numbers and of the numerical format that is chosen when completing the number sequence on the clock face.

All neuropsychological tests, indeed, involve multiple cognitive skills (there is not a single test that can be considered a pure measure of a specific cognitive ability; Pruneti et al., 2018) and knowledge about the cognitive abilities that a test (or an item of a test) aims to evaluate is critical for the correct ***interpretation*** of an individual’s performance on this test. In the case of global cognitive tests, such a knowledge can help the examiner to interpret not only a total score, but also the pattern of performance across different items. For example, Pasqualetti et al. (2002) highlight that, by only failing the MMSE memory items, one would obtain an MMSE score of 27, which is usually classified as normal. However, such failure could subtend a selective long-term memory impairment and signal preclinical conditions (i.e., an MCI of the amnesic type) that will evolve into dementia. A similar, apparently normal, score may thus call for further investigation.

Test users should then be, at the very least, supervised by an expert when tests are scored and results are interpreted. Even more crucially, expert supervision is required when the appropriate cognitive tests have to be ***selected***. Cognitive tests should be selected by people with in-depth knowledge of the psychometric properties of the tool to be used (i.e., its sensitivity, specificity, reliability, validity and predictive values). Only this kind of in-depth knowledge can lead to informed choices about what the most appropriate tools for different settings, populations and purposes are.

Indeed, the psychometric properties of a test are not fixed attributes but features of how it performs when it is applied to specific study samples (with a specific purpose), and are influenced by many factors that include age, occupation, education, and environmental context. In screening programs, different screening tools, or different cut-offs, may then be suitable depending on the target population and on the setting (community, primary, residential, or acute care) in which the screening is conducted. In particular, it is of great importance whether the screening will be conducted in selected elderly populations (e.g., people living in nursing homes, who usually need assistance for performing daily activities) or unselected populations. In the latter case, screening tests are more likely to produce false positives (e.g., Flicker et al., 1997) and tests with different sensitivities or different cut-off should be used in the two cases. Both the specificity and sensitivity of tests for dementia can be indeed affected by variations in its base rates in different populations (Ivnik et al., 2000). More generally, to adequately serve screening purposes, the selected tools should have been validated in population-based samples representing the target samples of the screening. In addition, the appropriate levels of sensitivity and specificity of the to-be-used test depend on its aims: population screening programs usually require higher cut-off scores than diagnostic settings (e.g., 28 instead of 24 for the MMSE), to minimize the risk of missing cases of cognitive impairment, but different cut-offs may also be appropriate according to the specific purposes of the screening (e.g., screening for dementia vs. MCI). In general, low specificity and high sensitivity may be appropriate when the screening is followed by a diagnostic workup in which many other cognitive tests are expected to be used. Knowledge of the psychometric properties of the selected screening tests is thus also critical in order to plan subsequent diagnostic phases.

For some screening tests, data are indeed available from different populations. MMSE scores, for example, are known to be influenced by age, education levels, social class, and sex (O’Connor et al., 1989; Lopez et al., 2005). Moreover, the test has been shown to have a major measurement limitation, that is, the frequent observation of either floor or ceiling effects when people with severe dementia or highly educated individuals with MCI are tested, respectively (Trzepacz et al., 2015). Accordingly, the MMSE may not be the most suitable instrument to use when samples from such populations are to be screened, and its cut-off should be changed to adjust sensitivity on the basis of the sample demographic characteristics, the number of false positives/negatives that are expected, whether the MMSE is the only screening instrument or other tools will be used to refine the outcomes (Pasqualetti et al., 2002; Tang-Wai et al., 2003; O’Bryant et al., 2008; Hoops et al., 2009).

Test selection may also imply the choice of a specific test version (with specific test instructions) which also depends on a neuropsychologically informed analysis of the purpose of the test (e.g., to unveil mild vs. severe impairment of certain cognitive functions) in that particular diagnostic setting. For example, it has been argued that the use of pre-drawn clocks in the CDT is useful to tap perceptual functions whereas free-drawn clocks place greater demands on language, memory, and executive functions (Freedman et al., 1994). Moreover, the request to draw the hands on the clock to indicate “10 past 11” (i.e., two numbers that are very close to each other in magnitude and on the clock face), as in Watson et al.’s (1993) version, places greater demands on executive functions (and results in a more difficult task) compared to when times on the hour or half-hour are requested (Tuokko et al., 1995).

### Differential Diagnosis

Once the suspicion of dementia is confirmed, most clinical practice guidelines recommend the referral to a specialist setting where a more detailed evaluation (requiring different specialist competencies and a multidisciplinary approach, cf., Pruneti et al., 2018) is conducted to perform a differential diagnosis in accordance with type-specific validated criteria (NICE, National Institute for Health and Clinical Excellence, 2018). This phase usually includes a comprehensive neuropsychological assessment (NPA) in which a crucial role is played by cognitive testing. Cognitive tests administered in this phase are generally specific to a particular set of cognitive abilities (i.e., each test is specifically designed to assess mainly certain cognitive functions), rather than being global cognitive tests. By evaluating performance across tests, in conjunction with the relevant medical history and behavioral observations, it is possible to define a specific diagnosis, assess the severity of impairment, and formulate a prognosis. Inclusion of NPA as part of the comprehensive investigation of this phase is indeed strongly recommended: it improves diagnostic accuracy over and above both routine clinical evaluation and (possible) laboratory/neuro-imaging tests (Geroldi et al., 2008).

It is widely recognized that NPA requires neuropsychological expertise in order to administer and score cognitive tests, as well as to interpret and communicate test results (cf., e.g., American Psychological Association, 2014). We would argue, however, that such an expertise may be very useful even when an in-depth NPA cannot be performed (e.g., because of time or resource constraints), and most of the information about the neuropsychological profile of the testee is collected through the same global cognitive tools that are designed for – and usually administered in – previous phases of the diagnostic process. Not only can these tools be helpful to obtain dichotomous findings (positive vs. negative outcomes in the screening or confirmatory phases) but, if adequately used, they can also provide more refined information that is useful for the differential diagnosis.

The MMSE, for example, can be a prominent source of information. Studies investigating its factorial structure have individuated two-to-three main factors underlying the MMSE score, each of which can be associated with different cognitive profiles that are typical of different types of dementia (see Noale et al., 2006; Shigemori et al., 2010).

As suggested by these studies, the factor including temporal/spatial orientation and delayed recall items is associated with episodic memory. It has been proposed that selectively poor performance on such items may be used as a marker of episodic memory impairment in early AD or MCI of the amnesic type: the summation of the scores obtained on these items indeed appears to be more strongly associated with AD, not only than the total MMSE score, but also than scores on specific tests of episodic memory (e.g., the Free and Cued Reminding Test) usually recommended for the detection of the amnestic syndrome in AD (Carcaillon et al., 2009). The factor including constructional praxis, reading, verbal comprehension and attention/concentration items is more linked to working memory abilities, whereas the factor including naming, verbal repetition, immediate memory, and writing items reflects verbal skills and consolidated (semantic) knowledge. Indeed, these items put less emphasis on episodic memory thus being less sensitive to the memory loss that is typical of AD. In contrast, performance on these items (or on some of them) may be more impaired in other forms of dementia. For example, Frontotemporal dementia (FTD) patients may show inattention and poor organization, as well as language impairment (the behavioral and language variants of FDT, respectively), in the face of a relative preservation of episodic memory (Wittenberg et al., 2008). An analysis of performance on items tapping executive functioning and language (see Table 1), and a comparison with performance on the orientation and delayed recall items, may help in the differential diagnosis between AD and FTD. The rate of progression of cognitive decline, as estimated by the changes in the MMSE score over time, can also provide helpful insight for such a differential diagnosis. Chow et al. (2006), for example, found that both patients with language and behavioral FTD show a more rapid progression of decline on the language MMSE items than AD patients and, vice versa, a slower progression on constructional praxis^2^.

The MoCA, too, can give useful information about possible impairments in distinct dimensions of cognitive functioning and, thus, for the differential diagnosis between different forms of dementia (Freitas et al., 2012). MoCA’s attention and executive sub-scores, for example, are composed of items that rely on cognitive functions associated with frontal lobe processing (see Table 1). They are considered to be particularly sensitive to frontal lobe dysfunctions (Nasreddine et al., 2005) and helpful in carrying out a differential diagnosis between AD and FDT (Coleman et al., 2016). Indeed, the MoCA includes critical subtests in which AD and FDT patients have been shown to present symmetrically different performances, that is items assessing verbal fluency and language production, as well as orientation and episodic memory (i.e., delayed recall). These items can be used to calculate the so-called VLOM ratio [(verbal-fluency + language)/(orientation + memory); Mathuranath et al., 2000], which can differentiate quite well between AD and FTD patients, with AD patients typically showing a higher ratio than FDT patients (i.e., relatively better performances on VL items compared to OM items; Larner, 2018).

The pattern of performance observed in the MoCA, and the possible differences between the testee’s performance on VL and OM items, should obviously be integrated with other sources of information, such as the testee’s behavior during test administration. It is well-known, indeed, that the most common form of FDT, that is, its behavioral variant, presents with an onset of symptoms characterized more by behavioral changes than cognitive deficits (e.g., stereotypical movements, compulsive-like behaviors), thus making the observation of testees’ behavior at least as important as their performance on neuropsychological tests (Rascovsky et al., 2011).

Qualitative, in addition to quantitative, assessment of testees’ performance is also very important: testees’ errors can be a useful source of information for the differential diagnosis if correctly interpreted. For example, Lee et al. (2009) showed that, in the CDT, AD patients usually make more errors revealing deficits in accessing knowledge of the features and meaning of a clock (e.g., the clock hands are absent or the time is simply written on the clock instead of being represented as a particular position of the hands on the clock) compared to patients with Parkinson’s disease dementia (PDD) or subcortical vascular dementia (VaD). On the contrary, PDD and VaD patients showed more errors reflecting a deficit in executive functioning, such as planning and perseverative errors (e.g., the patient draws three or more hands or a given number is written more than once on the clock).

Only in-depth familiarity with neuropsychological testing and knowledge of the aims and psychometric properties of the specific neuropsychological tools being administered may enable the examiner to perform these refined evaluations of testees’ performance. Such knowledge allows an informed, competent and flexible use of these tools and can make data obtained from global cognitive tests very helpful. These data can be indeed useful even when resources are available for performing a throughout NPA (i.e., when the differential diagnosis can also rely on the results of domain-specific neuropsychological tests), as they can orient the clinician toward a specific diagnosis to be confirmed through targeted diagnostic tools.

## Conclusion

Throughout this piece we have argued for the need of a synergy between the use of brief global cognitive tests and neuropsychological expertise in both detection and differential diagnosis of dementias. At the detection stage, neuropsychological expertise can make a real difference in the *selection, administration, scoring*, and *interpretation* of such tests, as well as in the *communication* of results both to other health care professionals involved in the detection process and to patients, their families and caregivers. Furthermore, neuropsychological expertise would enable the use of these tests to inform and orientate the differential diagnostic process at later stages, thereby saving time and efforts that can be used to help the patients and caregivers in the long process of knowledge and acceptance of dementia.

## Author Contributions

BT and MR drafted the manuscript. All authors were involved in the critical revision of the manuscript.

## Conflict of Interest

The authors declare that the research was conducted in the absence of any commercial or financial relationships that could be construed as a potential conflict of interest.

## Footnotes

1 According to the Clinical Neuropsychology Synarchy (Smith and CNS, 2019), the core competencies that are required of professionals working in clinical neuropsychology are knowledge of (a) general psychology (basic principles, problems, and methods underlying the science of psychology and main cognitive processes identified by research in experimental cognitive psychology and cognitive neuropsychology), (b) clinical psychology (including development of normal and abnormal behavior and cognition throughout the lifespan), (c) statistics and neuropsychological research methods, (d) neuropsychological assessment (current classification of neuropsychological symptoms, main tests used to detect such symptoms and their psychometric properties), (d) cultural and individual differences, (e) functional neuroanatomy and clinically relevant brain-behavior relationships, (e) neurological and related disorders (including their etiology, pathology, course and treatment), (f) neuroimaging and other neurodiagnostic techniques (g) professional ethics, (h) neuropsychological intervention, including treatment and rehabilitation. In addition, skills in the following domains are required: (a) decision-making and diagnosis, (b) consultation (patients, families, medical colleagues, agencies, etc.) and communication of both evaluation results and recommendations to diverse audiences, (c) information gathering, (d) teaching and supervision (e) continuous updating of relevant knowledge through reviews of critical literature (see also Hessen et al., 2018).

2 It is worth noticing that atypical forms of AD (i.e., AD forms with non-amnestic presentation) have been described that can mimic other forms of dementia. Among these AD variants, there are very rare forms of AD that present with executive and behavioral deficits, together with disproportionate frontal lobe atrophy, and can be easily mistaken with FDT (Ljubenkov and Geschwind, 2016). The majority of the atypical forms of AD present with an early-onset (they present in people younger than 65). In such early-onset cases, structural neuroimaging and cerebrospinal fluid examinations are usually performed, as recommended by the American Academy of Neurology and European Federation of Neurological Societies guidelines (Knopman et al., 2001). Laboratory investigations, along with clinical assessment, may help in clarifying the picture (Rossor et al., 2010). It should be noted, however, that the description of these rare AD forms has been limited to few case reports and studies with small series of patients. Therefore, clinical and neuropathological characteristics of these forms, including their evolution over time, are not yet well understood (Ossenkoppele et al., 2015). At the moment, there are no clear neuropsychological indexes that can help distinguish between FDT and these behavioral/executive variants of AD.
